# Pseudo-T2 mapping for normalization of T2-weighted prostate MRI

**DOI:** 10.1007/s10334-022-01003-9

**Published:** 2022-02-12

**Authors:** Kaia Ingerdatter Sørland, Mohammed R. S. Sunoqrot, Elise Sandsmark, Sverre Langørgen, Helena Bertilsson, Christopher G. Trimble, Gigin Lin, Kirsten M. Selnæs, Pål E. Goa, Tone F. Bathen, Mattijs Elschot

**Affiliations:** 1 Department of Circulation and Medical Imaging, Norwegian University of Science and Technology, Olav Kyrres Gate 9, 7030 Trondheim, Norway; 2 Department of Radiology and Nuclear Medicine, St. Olavs Hospital, Trondheim University Hospital, Trondheim, Norway; 3 Department of Cancer Research and Molecular Medicine, Norwegian University of Science and Technology, Trondheim, Norway; 4 Department of Urology, St. Olavs Hospital, Trondheim University Hospital, Trondheim, Norway; 5 Department of Medical Imaging and Intervention, Chang Gung Memorial Hospital at Linkou and Chang Gung University, 5 Fuhsing St., Guishan, Taoyuan, 33382 Taiwan; 6 Department of Physics, Norwegian University of Science and Technology, Trondheim, Norway

**Keywords:** Prostate, Prostatic neoplasms, Medical image processing, Magnetic resonance imaging, Multicenter study

## Abstract

**Objective:**

Signal intensity normalization is necessary to reduce heterogeneity in T2-weighted (T2W) magnetic resonance imaging (MRI) for quantitative analysis of multicenter data. AutoRef is an automated dual-reference tissue normalization method that normalizes transversal prostate T2W MRI by creating a pseudo-T2 map. The aim of this study was to evaluate the accuracy of pseudo-T2s and multicenter standardization performance for AutoRef with three pairs of reference tissues: fat/muscle (AutoRef_F_), femoral head/muscle (AutoRef_FH_) and pelvic bone/muscle (AutoRef_PB_).

**Materials and methods:**

T2s measured by multi-echo spin echo (MESE) were compared to AutoRef pseudo-T2s in the whole prostate (WP) and zones (PZ and TZ/CZ/AFS) for seven asymptomatic volunteers with a paired Wilcoxon signed-rank test. AutoRef normalization was assessed on T2W images from a multicenter evaluation set of 1186 prostate cancer patients. Performance was measured by inter-patient histogram intersections of voxel intensities in the WP before and after normalization in a selected subset of 80 cases.

**Results:**

AutoRef_FH_ pseudo-T2s best approached MESE T2s in the volunteer study, with no significant difference shown (WP: *p* = 0.30, TZ/CZ/AFS: *p* = 0.22, PZ: *p* = 0.69). All three AutoRef versions increased inter-patient histogram intersections in the multicenter dataset, with median histogram intersections of 0.505 (original data), 0.738 (AutoRef_FH_), 0.739 (AutoRef_F_) and 0.726 (AutoRef_PB_).

**Discussion:**

All AutoRef versions reduced variation in the multicenter data. AutoRef_FH_ pseudo-T2s were closest to experimentally measured T2s.

**Supplementary Information:**

The online version contains supplementary material available at 10.1007/s10334-022-01003-9.

## Introduction

Cancer is expected to rank as the leading cause of death in the twenty-first century, with the burden of cancer incidence and mortality rapidly growing worldwide [1]. Among men, prostate cancer is the second most frequently diagnosed cancer in the world, and the leading cause of cancer death in 48 countries [1].

The diagnosis of prostate cancer is initiated by prostate-specific antigen measurements and determination of clinical stage with digital rectal examinations [2]. The final diagnosis is based on the microscopic evaluation of prostate tissue obtained via needle biopsy [3]. With recent technological advancements and growing availability, multiparametric magnetic resonance imaging (mpMRI) is increasingly being used in the detection, staging and treatment planning of prostate cancer [2]. MpMRI combines conventional anatomical T2-weighted (T2W) MRI pulse sequences with functional MRI pulse sequences [4], providing a non-invasive assessment of multiple physiological parameters such as vascularization and cellularity [5, 6].

The Prostate Imaging-Reporting and Data System (PI-RADS) is designed to promote standardization and minimize variation in the acquisition, interpretation and reading of mpMRI [7], where the use of T2W images (T2WI) has mainly been limited to qualitative evaluation of prostate anomalies. Its utility for quantitative analysis is hindered by non-standard signal intensities attributed to MRI scanner parameters such as the field strength, coil type, signal amplification and acquisition protocols [8–10]. Thus, signal intensity normalization of T2WI is required for quantitative analysis, and to enable inter- and intra-patient comparison. Signal intensity normalization is also paramount for the development of robust MRI-based computer aided diagnosis of prostate cancer based on machine learning techniques [11].

While signal intensities may vary, the intrinsic tissue T2 relaxation times are expected to be independent of the hardware, as they reflect the absolute relaxation of the nuclei regardless of their relative position to the coil [12]. T2s are also comparatively field independent from 1.5 to 3T [13]. Quantitative T2 imaging of the prostate has shown high reproducibility [14, 15], and the T2s have been shown to vary significantly between prostate cancer and normal gland tissue [12, 15]. However, currently available T2 mapping techniques are primarily based on spin echo relaxometry strategies that suffer from lengthy acquisition times. Fast T2 mapping techniques such as those based on dictionary matching have been proposed, but are not yet widely available and/or validated for clinical prostate imaging [16–19], and quantitative T2 imaging is not included in the standardized clinical pathway following PI-RADS [7].

AutoRef is a recently developed automated method for prostate T2WI normalization using a pair of reference tissues (fat and muscle) [20]. During normalization, the T2WI are rescaled to resemble the tissue T2s, and are hence named pseudo-T2 maps. The aim of this study was to evaluate the accuracy of pseudo-T2s in the prostate produced by AutoRef with three pairs of reference tissues, and their normalization performance on multicenter data. The method was, therefore, applied on T2WI of asymptomatic volunteers with experimentally measured prostate T2s and prostate cancer patients from a large, multicenter dataset.

## Materials and methods

### Subjects

A study on volunteers was performed to measure reference tissue T2s and to validate the accuracy of AutoRef pseudo-T2s on a cohort with measured prostate T2s. Eight asymptomatic volunteers (median age 28.5, range 26–65 years) were recruited for this purpose. The Regional Committee for Medical and Health Research Ethics (REC Central Norway) approved the study, and all volunteers signed informed consents prior to recruitment (REC identifier 2014/1289).

Multicenter data were used to train the AutoRef automated reference tissue detection and to evaluate the normalization performance of AutoRef on a large, heterogeneous dataset. A summary of the origins and usage of the multicenter T2WI is found in Table 1. The institutional review board at Chang Gung Memorial Hospital approved the protocol of this study (Chang Gung Medical Foundation IRB 201901295B0). Informed consent was waived because of the retrospective nature of the study and the analysis used anonymous clinical data. The use of the in-house data was approved by the institutional review board and The Regional Committee for Medical and Health Research Ethics (REC Central Norway, identifier 2017/576, 2013/1869). All in-house patients signed informed consents prior to the initiation of the study. The remaining data came from publicly available datasets [21, 22].

**Table 1:** Details about the datasets used for training reference tissue detectors and evaluate the AutoRef normalization method

Cohort	Origin	Scanner (number of patients)	Total number of patients	Median age (range)	Acquisition dates	Usage in AutoRef
In-house	St. Olavs hospital, Trondheim University Hospital, Norway	3T Magnetom Skyra (339) and 3T Magnetom Biograph mMR (28) from Siemens Healthineers	367	66 (45–79)	May 2014–Dec. 2018	Train (*n* = 20), evaluate (*n* = 347)
Prostate X	Radboud University Medical Centre, the Netherlands	3T Magnetom TrioTim (57) and 3T Skyra (286) from Siemens Healthineers	343	66 (48–83)	2012	Train (*n* = 20), evaluate (*n* = 323)
CGMH	Linkou Chang Gung Memorial Hospital, Taiwan	3T Magnetom Biograph mMR (8), TrioTim (23) and Skyra (18) from Siemens Healthineers; 3T Discovery MR750 (278) and 1.5T Optima MR450w (179) from GE Healthcare; 3T Ingenia (10) from Philips Healthcare	516	69 (45–95)	Feb. 2014–Dec. 2017	Evaluate
Promise 12	University College London, United Kingdom and Radboud University Medical Centre, the Netherlands	Siemens Healthineers (1.5T and 3T, all endorectal coil cases excluded)	39	N/A	N/A	Train

The full set consisted of T2-weighted images from St. Olavs hospital (in-house), Chang Gung Memorial Hospital (CGMH) and the publicly available datasets Promise 12 [17] and Prostate X [18]

### Data acquisition for asymptomatic volunteers

MR images were acquired for the eight asymptomatic volunteers on a Magnetom Skyra 3T MRI system (Siemens Healthineers, Erlangen, Germany) at St. Olavs hospital, Trondheim University Hospital, Norway. Transversal MR images, covering the whole prostate, were acquired with a combination of a 16-channel body matrix coil and 1–2 coil elements from a table integrated 32-channel spine coil. A multi-echo spin echo (MESE) pulse sequence (repetition time (TR): 2120–2450 ms, number of slices: 7–11, resolution matrix: 256 × 256, field of view (FoV): 250 mm × 250 mm, slice thickness: 4 mm, slice gap: 50 percent) was acquired to measure the T2s of the prostate and surrounding reference tissues: muscle, pelvic bone, femoral heads (only the yellow bone marrow) and fat. The variations in TR and number of slices had to be made due to inter-subject variations in specific absorption rate. The MESE sequence was applied with 17 echoes with TEs ranging from 10.6 to 180.2 ms. The Generalized Autocalibrating Partial Parallel Acquisition (GRAPPA) technique [23] was applied with an acceleration factor of 2, giving the MESE sequence a total acquisition time of 07:33 min. A T2W turbo spin echo sequence (TR: 5330 ms, TE: 104 ms, flip angle: 160°, number of slices: 26, resolution matrix 384 × 384, FoV: 192 mm × 192 mm and slice thickness: 3 mm) was acquired for seven of the volunteers. This sequence had an acquisition time of 05:43 min.

**Fig. 1:**
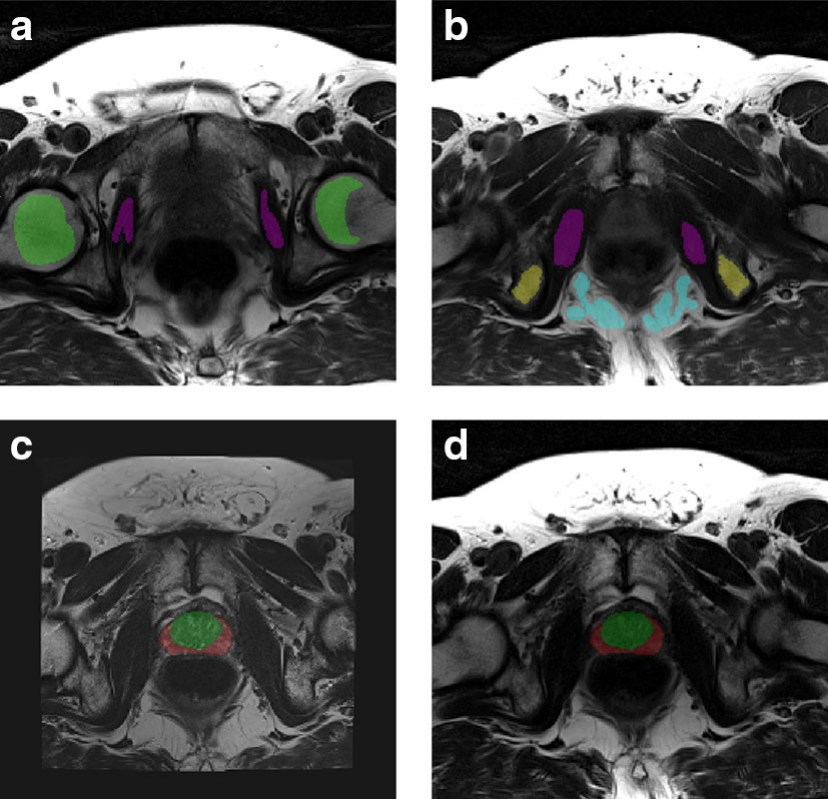
**a**, **b** Two slices of the transversal multi-echo spin echo (MESE) image (TE = 106 ms) of an asymptomatic volunteer, with manual delineations within the reference regions. Purple indicates the obturator internus muscle, yellow the ischial tuberosity (pelvic bone), blue the ischioanal fossa (fat) and green the yellow bone marrow in the femoral heads. **c** Transversal T2-weighted image registered to the MESE image space, with co-registered prostate segmentation. The peripheral zone is red, while the remaining zones (transitional zone, central zone and anterior fibromuscular stroma) are green. **d** Transversal MESE image (TE = 106 ms) with registered manual prostate segmentations

Regions of interest (ROIs) were manually drawn (K.I.S.) within the reference tissues on the MESE images, as shown in Fig. 1. ROIs were drawn on all image slices containing these tissues, but the most superior slice was excluded as it appeared to have higher relative signal intensity. Manual segmentations of the whole prostate gland (WP), peripheral zone (PZ) and remaining zones (transitional zone (TZ), central zone (CZ) and anterior fibromuscular stroma (AFS)) were delineated on the T2WI by a radiology resident (E.S.) under the supervision of a radiologist with more than 10 years′ experience in prostate imaging (S.L.), using ITK-SNAP [24]. To obtain the prostate segmentations on the MESE images, the T2WI were registered to the MESE images using Elastix v 4.9.0 [25], and the segmentations were transformed accordingly. Image registration parameters can be found in Online Resource 1.

A least-squares monoexponential fit of the change in mean ROI intensities (*I)* with TE, following the equation

1 $$I\left( {{\text{TE}}} \right) = I_{0} e^{{ - \frac{{{\text{TE}}}}{{T_{2} }}}} ,$$

provided estimates of the T2s in the reference tissues and phantom samples [26]. To get the prostate T2 values, a T2 map was created by a robust fitting of Eq. 1 with bi-square weights in each voxel, with interval restrictions [0, 500] for both *I*_0_ and T2. Only fits of voxels with coefficient of determination *R*^2^ ≥ 0.85 were kept for further analysis. Because of signal contamination due to stimulated and indirect echoes generated in the MESE sequence [27], only data from the first five even numbered echoes (with TEs of 21.2, 42.4, 63.6, 84.8 and 106.0 ms) were included in the fitting. The influence of non-monoexponential decays and noise were expected to increase with increasing TE, and hence only the early echoes were included.

The accuracy of the T2s measured by MESE was evaluated on a phantom of ten samples with known T2s estimated by various concentrations of MnCl_2_ in the samples. All details are provided in Online Resource 2.

### Acquisition of multicenter data

A study on multicenter data was conducted to evaluate the normalization performance of AutoRef on T2WI of various origins, and whole prostate segmentations were needed for this purpose. The in-house manual segmentations of the whole prostate were performed by a radiology resident (E.S.) under the supervision of a radiologist (S.L.) at St. Olavs hospital. The segmentations for the Prostate X dataset were performed by imaging experts with more than 25 years′ combined expertise in prostate imaging and reviewed by radiation oncologists at Miller School of Medicine, Miami, FL, USA.

Manual prostate segmentations were lacking from the CGMH set, and these images were therefore segmented automatically with a model based on 3D nnU-Net v 1.5 [28] trained on a combination of data from Prostate X (*n* = 200), Promise 12 (*n* = 50) and in-house cases from the 3T Magnetom Skyra (*n* = 220). The network training, validation and testing were performed on a single NVIDIA Tesla P100 PCIe 16 GB GPU in Ubuntu 18.04.4 LTS system. The network was implemented with PyTorch (version 1.4.0) using Python (version 3.6.9). Poor prostate segmentations were excluded based on an in-house developed segmentation quality control system [29], where only segmentations with a quality score above or equal to 85/100 were included.

The AutoRef training set differed from the automated segmentation training set, and is described in Table 1. It consisted of T2WI from 79 patients from various centres and scanners: 39 cases from Promise 12 [21], 10 cases from Prostate X [22] acquired on a 3T Magnetom Skyra scanner, 10 cases from Prostate X [22] acquired on a 3T Magnetom TrioTim scanner and 20 cases from the in-house dataset acquired on a 3T Magnetom Skyra scanner. These cases were excluded from the evaluation dataset. The AutoRef evaluation dataset consisted initially of T2WI from 13 MRI scanners located at three different institutions. Only MRI acquired before prostate cancer treatment were included, and when a patient had pretreatment MRI acquired at multiple time points only the first scan was included. 200 cases (out of 721 eligible) were excluded from the CGMH cohort due to poor automated prostate segmentations. The remaining number of patients from each scanner ranged from 1 to 319. For practical purposes, only scanners with more than five patient scans were included—leading to the exclusion of three scanners (*n* = 6 patients in total). The final evaluation set thus consisted of 1186 pretreatment T2WI from 1186 prostate cancer patients, providing variations in MRI scanner models, manufacturers, field strengths and acquisition protocols (Table 2).

**Table 2:** Variation in acquisition parameters listed for each MRI scanner in the multicenter evaluation set

Scanner	Field strength (T)	Repetition time (ms)	Echo time (ms)	Slice thickness (mm)	In-plane resolution	Flip angle	T2WI protocol
St. Olavs Skyra	3	4100–10,120	101–108	3–3.5	0.5 × 0.5–0.6 × 0.6	145–160	TSE
Prostate X Skyra	3	3880–8624	101–112	3–4.5	0.3 × 0.3–0.6 × 0.6	156–160	TSE
CGMH Discovery MR750	3	3892–12,753	54–144	3–4	0.35 × 0.35–0.43 × 0.43	142	PROPELLER
CGMH Optima MR450w	1.5	4499–7765	106–126	3–4	0.31 × 0.31–0.39 × 0.39	160	PROPELLER
Prostate X TrioTim	3	4000–5870	101–103	3–5	0.56 × 0.56–0.70 × 0.70	120–150	TSE
St. Olavs Biograph mMR	3	6840	104	3	0.5 × 0.5	147–160	TSE
CGMH TrioTim	3	3913–4240	92–102	3–4	0.40 × 0.40–0.56 × 0.56	140	TSE
CGMH Skyra	3	5800–7200	101	4	0.63 × 0.63–0.69 × 0.69	160	TSE
CGMH Ingenia	3	4279–4636	90	4	0.35 × 0.35–0.39 × 0.39	90	TSE
CGMH Biograph mMR	3	3600	78–89	4	0.35 × 0.35–0.47 × 0.47	150	TSE

*T2WI* T2-weighted image, *TSE* turbo spin-echo, *PROPELLER* periodically rotated overlapping parallel lines with enhanced reconstruction

### The normalization method

AutoRef has previously been described by Sunoqrot et al. [20], where the underlying assumption is that inherent T2 relaxation times of reference tissues remain approximately constant across patients and MRI systems. The method utilizes a pair of automatically detected reference tissues, with one tissue of longer T2 than the prostate and one of shorter. In our current study, three pairs of reference tissues have been evaluated: the obturator internus muscle (referred to as muscle) was the only chosen reference tissue of lower T2 and T2W intensity than the prostate, and therefore, used in all reference tissue pairs. It was paired with either ischioanal fossa (referred to as fat, AutoRef_F_), ischial tuberosity (referred to as pelvic bone, AutoRef_PB_) and the yellow bone marrow in the femoral heads (referred to as femoral head, AutoRef_FH_). The reference tissues were chosen based on their expected T2s, their potential to be automatically detected and delineated, and whether they are within the field of view in standard prostate T2WI. Of note, AutoRef_F_ has been investigated in previous work [20], while AutoRef_FH_ and AutoRef_PB_ are new methods.

An aggregate channel features object detector (acfObjectDetector, Matlab R2019b, MathWorks, Natick, MA, USA) was trained to set rectangular ROIs surrounding each reference tissue on the 2D transverse T2WI slices. To train the reference tissue detectors, rectangular ROIs were manually drawn around reference tissues on the T2WI of the AutoRef training set, where each tissue was marked on three slices (when available) per acquired MRI. The procedure and parameters for training the tissue detectors were kept as reported in [20], besides the number of iterative training stages (changed from 3 to 5). Tissue detector focus regions for muscle and fat were kept as in [20], while focus regions for the new reference tissues were set based on where they were expected to be: the anterior 75% of image rows and middle (25–75%) of slices for the femoral heads, and the posterior 75% of rows and inferior 50% of slices for the pelvic bone.

To normalize the T2WI, the images were first pre-processed with N4 bias field correction [30] and rescaled to the 99th percentile intensity value, and the transverse slices were resized to 384 × 384 pixels with 0.5 × 0.5 mm in-plane resolution, all according to [20]. Rectangular ROIs were then automatically detected around all reference tissues by the reference tissue object detectors, and ROIs were further processed by extracting the largest connected structure within the region. This was achieved by Otsu thresholding [31], in accordance with [20], and morphological opening with a disk shape of three-pixel radius (in comparison to the one-pixel radius used in [20]). This resulted in automatically detected and delineated reference tissues.

The entire 3D T2WI were then normalized by linearly scaling the 10th percentile of muscle tissue intensity (marked low) and 90th percentile of the paired reference tissue intensity (fat, pelvic bone or femoral head; marked high) to their corresponding T2s. The percentiles were used instead of median or mean intensity due to potential inaccuracies in the automatic delineation of the reference tissues, and the reference T2s used were the reference tissue T2s measured in the volunteers. The linear scaling of the T2WI followed the equation

2 $${\text{pseudo}} {-} T_{2} \left( {x,y,z} \right) = \frac{{I\left( {x,y,z} \right) - I^{{{\text{low}}}} }}{{I^{{{\text{high}}}} - I^{{{\text{low}}}} }} \times \left( {T_{2}^{{{\text{high}}}} - T_{2}^{{{\text{low}}}} } \right) + T_{2}^{{{\text{low}}}} ,$$

where $I^{{{\text{high}}}}$ and $I^{{{\text{low}}}}$ were the 90th and 10th percentile, respectively. Equation 2 is constructed so that inserting a pixel intensity $I\left( {x,y,z} \right)$ equal to $I^{{{\text{low}}}}$ will give a pseudo-T2 equal to $T_{2}^{{{\text{low}}}}$, and inserting $I\left( {x,y,z} \right)$ equal to $I^{{{\text{high}}}}$ will give a pseudo-T2 equal to $T_{2}^{{{\text{high}}}}$. Any pixel intensities between the low- and high-intensity reference tissue will thus be scaled to a pseudo-T2 between $T_{2}^{{{\text{low}}}}$ and $T_{2}^{{{\text{high}}}}$, and the normalized images are therefore called pseudo-T2 maps.

### Evaluation of the normalization method

AutoRef normalization with all three pairs of reference tissues was applied to the seven T2WI of the asymptomatic volunteers. This enabled a comparison between generated pseudo-T2 and T2 calculated from the MESE images in the whole prostate gland and in the prostate zones. The paired Wilcoxon signed-rank test was used to test for differences between pseudo-T2s and MESE T2s, with *p* values less than 0.05 considered statistically significant.

In [20], AutoRef_F_ was compared to three other automated histogram-based normalization methods commonly used in the literature. It was then proven to be the overall best performing method, followed by Gaussian kernel normalization [32]. For further validation of AutoRef in the multicenter dataset, Gaussian kernel normalization was therefore evaluated in comparison with AutoRef. All T2WI used in the multicenter evaluation were pre-processed with N4 bias field correction [30].

Gaussian kernel normalization and the three versions of AutoRef were applied on all T2WI of the multicenter evaluation set (*n* = 1186), and the mean prostate intensity before and after normalization was calculated for each T2WI. Histograms of the mean prostate intensities in the multicenter dataset before and after normalization were assessed to evaluate the effect of normalization.

Histogram intersections of whole prostate voxel intensities were also used as a measure of multicenter normalization performance: Due to the large sample size, eight cases were randomly chosen from each of the ten scanners in the multicenter dataset, giving an evaluation subset of 80 cases. An equal number of cases from each scanner was selected to avoid bias, and the number eight was chosen as this was the lowest number of patients from one scanner. For each patient, histograms of the whole prostate voxel intensities were created for the original and each normalized T2WI. The intersected histogram area between all possible combinations of two patients in the evaluation subset was calculated before and after normalization, as described in [20]. The intersected areas could be between 0 and 1, where 1 would indicate two fully overlapping histograms and hence identical prostate intensity distributions between two patients.

The Mann–Whitney *U* test was applied to test for a significant difference between the prostate pseudo-T2s for each combination of AutoRef versions (i.e., AutoRef_F_‒AutoRef_FH_, AutoRef_F_‒AutoRef_PB_ and AutoRef_FH_‒AutoRef_PB_). Mann–Whitney *U* test was also applied to test for significant differences in prostate intensities in scanner pairs, for all normalization methods and the un-normalized images. With ten scanners, this test was applied on all the 45 possible scanner pairings.

## Results

### Asymptomatic volunteers

The reference tissue T2 relaxation times obtained from MESE, averaged over eight volunteers, were: 137.0 ± 2.7 ms for femoral head, 37.4 ± 0.9 ms for muscle, 98.7 ± 7.6 ms for pelvic bone and 129.7 ± 1.9 ms for fat.

**Fig. 2:**
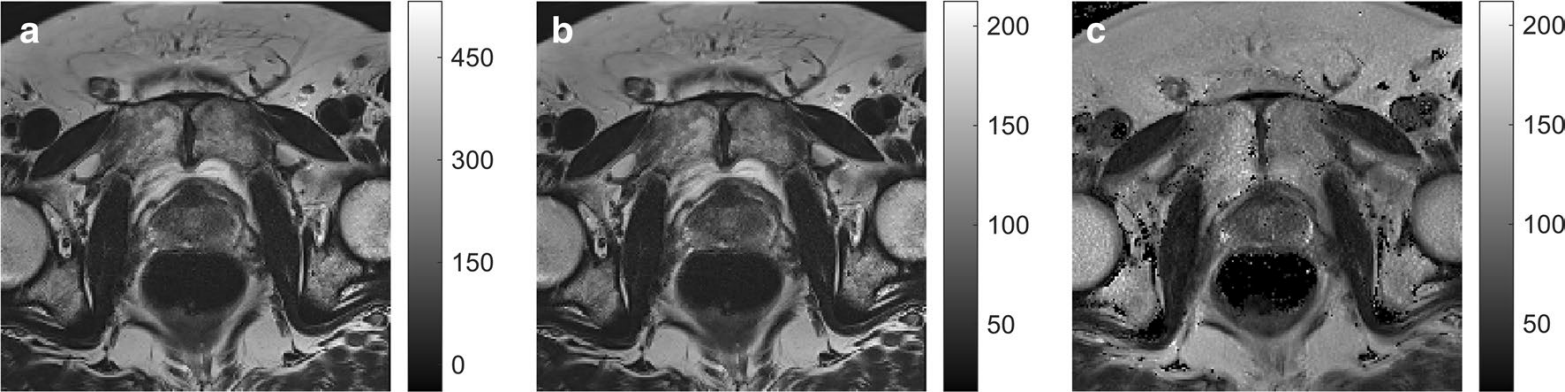
**a** Original bias field corrected T2-weighted image (T2WI), **b** AutoRef_FH_ pseudo-T2 map and **c** multi-echo spin echo (MESE) T2 map for one volunteer. The original T2WI and the pseudo-T2 map were window levelled from their minimum to maximum image intensity, while the MESE T2 map was kept on the same level as the pseudo-T2 map ([16 ms, 212 ms]). The T2WI was registered to the MESE image space before normalization

Table 3 shows the mean MESE T2s and AutoRef pseudo-T2s in the prostate zones, with *p* values from the paired Wilcoxon signed-rank test and mean absolute differences with standard deviations. AutoRef_FH_ was the only version where no significant differences were found between pseudo-T2s and MESE T2s in all zones, and was the method producing the highest pseudo-T2s. The similarity between prostate intensities of an AutoRef_FH_ pseudo-T2 map and a MESE T2 map can be seen in Fig. 2, with the original bias field corrected T2WI for comparison.

**Table 3:** The measured prostate T2 relaxation times with standard deviations from the multi-echo spin echo (MESE) imaging sequence and the prostate pseudo-T2s from AutoRef with different reference tissue pairs, averaged over seven volunteers

	MESE	AutoRef_FH_			AutoRef_F_			AutoRef_PB_		
	T2 (ms)	pT2 (ms)	*p* value	MD (ms)	pT2 (ms)	*p* value	MD (ms)	pT2 (ms)	*p* value	MD (ms)
PZ	87.4 ± 6.9	85.2 ± 5.5	0.69	2.2 ± 8.2	81.8 ± 4.9	0.11	5.6 ± 8.0	79.4 ± 6.7	< .05	8.0 ± 8.3
TZ, CZ and AFS	71.5 ± 3.7	68.9 ± 3.4	0.22	2.6 ± 4.2	66.7 ± 2.7	< .05	4.8 ± 3.8	65.2 ± 5.1	< .05	6.3 ± 5.5
Whole prostate	78.7 ± 4.9	76.1 ± 4.3	0.30	2.5 ± 5.7	73.4 ± 3.4	0.08	5.3 ± 5.4	71.6 ± 6.2	< .05	7.1 ± 6.5

Mean absolute differences (MD) between respective pseudo-T2s and MESE T2s are reported with standard deviations. All AutoRef versions used muscle as low-intensity reference tissue, and high-intensity reference tissues were: AutoRef_FH_: femoral head, AutoRef_F_: fat and AutoRef_PB_: pelvic bone

*PZ* peripheral zone, *TZ* transitional zone, *CZ* central zone, *AFS* anterior fibromuscular stroma, *pT2* Pseudo-T2

*p* values reported are from the paired Wilcoxon signed-rank test, testing for difference between AutoRef pseudo-T2s and the MESE T2s

### Multicenter evaluation

**Fig. 3:**
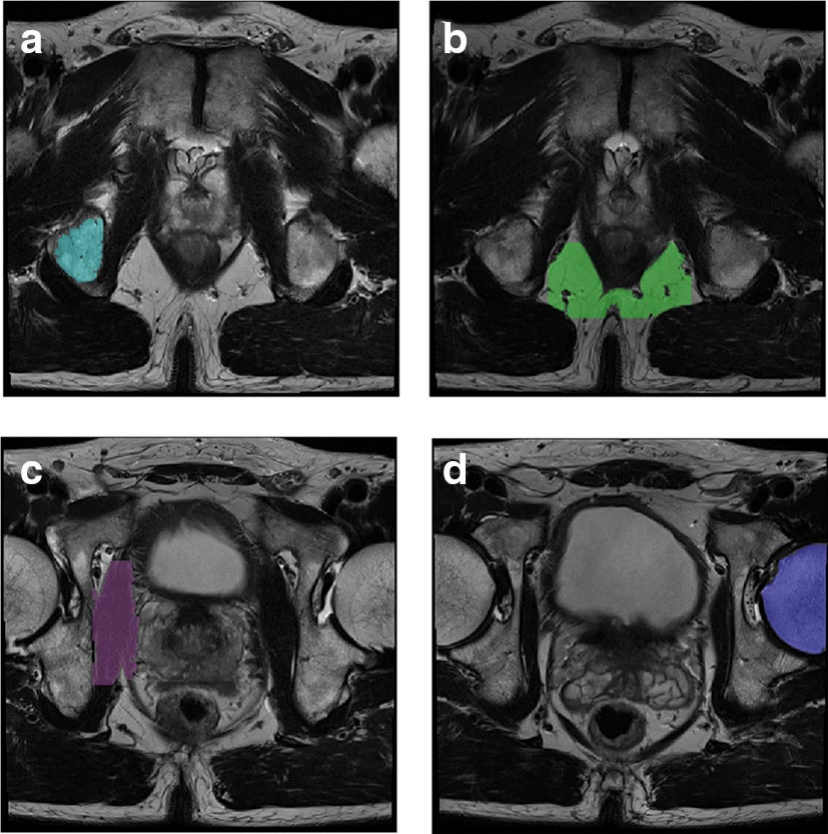
Reference tissues automatically detected and delineated in AutoRef for one patient from the publicly available dataset Prostate X [22]. **a** is the pelvic bone, **b** is the fat, **c** is the muscle and **d** is the femoral head

An example of a set of detected reference tissues is shown in Fig. 3. Mean scanner prostate pseudo-T2s from all three AutoRef versions applied on the multicenter evaluation set are reported in Table 4, indicating a reference tissue dependency on the pseudo-T2 similar to what was seen for the volunteers: AutoRef_PB_ provided the lowest pseudo-T2, and AutoRef_FH_ the highest. The Mann–Whitney *U* test showed a significant difference between the pseudo-T2s between all AutoRef versions (*p* < 0.001).

**Table 4:** Mean prostate pseudo-T2 with standard deviation after all three versions of AutoRef normalization

Scanner	Pseudo-T2 (ms)			Failed muscle	Failed fat	Failed femoral head	Failed pelvic bone	Number of patients
	AutoRef_F_	AutoRef_FH_	AutoRef_PB_					
St. Olavs Skyra	84.7 ± 7.7 (3)	88.0 ± 8.1 (0)	72.3 ± 7.2 (0)	0	3	0	0	319
Prostate X Skyra	81.6 ± 7.0 (1)	84.5 ± 7.5 (1)	70.7 ± 6.3 (0)	0	1	1	0	276
Prostate X TrioTim	84.1 ± 7.8 (1)	89.5 ± 7.3 (0)	73.2 ± 7.3 (0)	0	1	0	0	47
St. Olavs Biograph mMR	80.0 ± 7.4 (0)	81.2 ± 6.6 (0)	67.1 ± 5.3 (0)	0	0	0	0	28
CGMH Discovery MR750	87.3 ± 6.7 (10)	92.8 ± 7.3 (3)	72.1 ± 5.2 (1)	1	10	2	0	278
CGMH Ingenia	78.7 ± 7.3 (0)	84.5 ± 8.6 (0)	72.9 ± 8.2 (0)	0	0	0	0	10
CGMH Optima MR450w	75.0 ± 6.4 (16)	78.2 ± 7.0 (22)	66.4 ± 6.3 (16)	16	2	15	0	179
CGMH Biograph mMR	72.0 ± 5.0 (0)	75.0 ± 4.7 (0)	62.7 ± 5.1 (0)	0	0	0	0	8
CGMH TrioTim	83.0 ± 6.7 (1)	88.1 ± 7.7 (0)	71.8 ± 6.7 (0)	0	1	0	0	23
CGMH Skyra	82.5 ± 7.6 (0)	86.0 ± 7.5 (0)	71.5 ± 7.1 (0)	0	0	0	0	18
Entire dataset	82.8 ± 8.1 (32)	86.7 ± 8.9 (26)	70.9 ± 6.7 (17)	17	18	18	0	1186

The number of patients where normalization failed due to lack of either reference tissue is reported in parenthesis

Table 4 also lists the number of cases where the object detector failed, establishing pelvic bone as the most stably detected high-intensity reference tissue with no failed cases. Second to pelvic bone came the muscle detector, where all but one failed case came from a single scanner (Optima MR450w). Fat and femoral head both failed in 18/1186 cases (1.5%), but the failed femoral head cases were to a larger degree overlapping with the failed muscle cases.

Optima MR450w was the only 1.5 T scanner of the evaluation set, which could explain the abundance of failed tissue detector cases from this scanner. Although the tissue detectors’ training set consisted of some 1.5 T cases, it mostly consisted of 3T cases—which also is the recommended choice for prostate cancer detection scans [7]. This could indicate that AutoRef performs best on cases from 3T MRI scanners, although a 91% success rate for the muscle detector and 92% for the femoral head detector on the 1.5 T evaluation cases is still deemed acceptable. Alongside Discovery MR750, Optima MR450w was also the only scanner utilizing a periodically rotated overlapping parallel lines with enhanced reconstruction (PROPELLER) T2W sequence, which is another possible explanation for the abundance of failed cases from these two scanners.

To enable direct comparisons between all the normalization methods in the analyses presented below, all cases where the reference tissue detector had failed in any of the AutoRef versions were excluded. The total number of excluded cases was 41, meaning that the reported pseudo-T2s are based on a set consisting of 1145 T2WI in total.

The scanner mean prostate pseudo-T2 differed between scanners. For example, the range in mean pseudo-T2 for AutoRef_FH_ was from 75.0 ± 4.7 ms (CGMH Biograph mMR) to 92.8 ± 7.3 ms (CGMH Discovery MR750). This difference could be caused by variations in acquisition protocols that the normalization procedure could not fully handle, or there could be biological differences between patient cohorts. Prostate cancer and benign abnormalities such as chronic prostatitis, atrophy, scars and hyperplasia are all shown to influence the T2W signal intensity [2], and thus some variation in mean prostate pseudo-T2 between patients is expected.

**Fig. 4:**
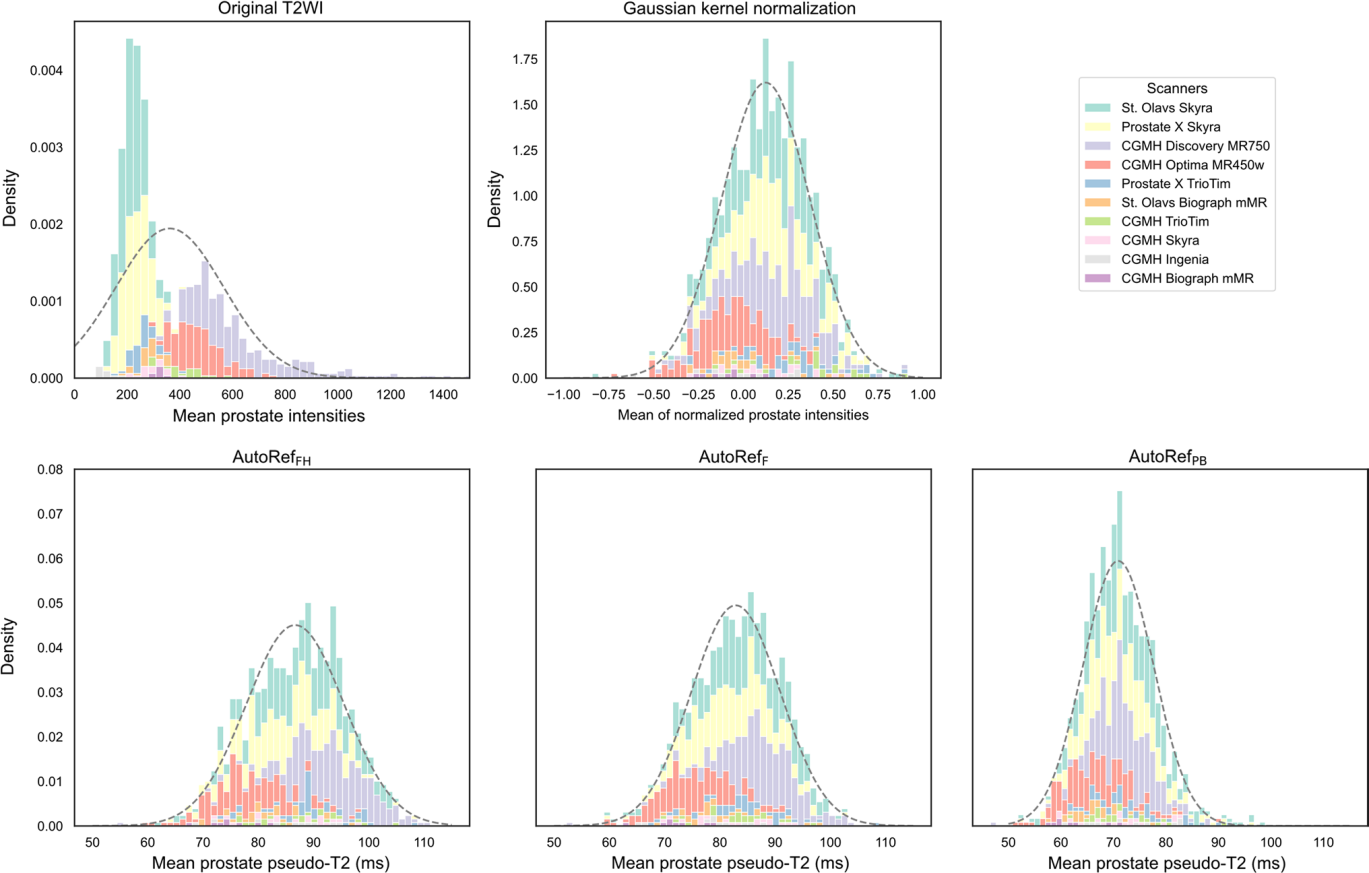
Histograms of mean prostate intensities in the multicenter cohort, with fitted normal distributions. The contributions from each MRI scanner are coloured and stacked. The histograms from the three AutoRef methods are on the same scale. *T2WI* T2-weighted image

Figure 4 shows the histogram of mean prostate intensities in the multicenter dataset, with accumulative contributions from each scanner coloured. It can be observed that normalization reduced scanner dependencies and made the mean prostate intensities approach a normal distribution. In addition, Mann–Whitney *U* tests showed that 42 out of 45 scanner pairs had significant different median whole prostate intensities for the original data, whereas this was reduced for Gaussian kernel normalization (27/45), AutoRef_F_ (26/45), AutoRef_FH_ (30/45), and AutoRef_PB_ (24/45).

**Fig. 5:**
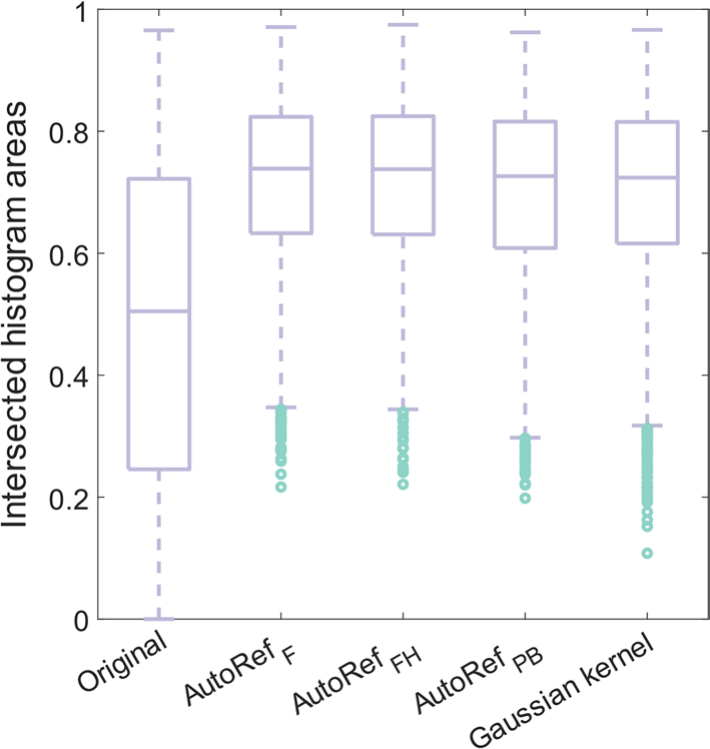
Boxplots of the intersected histogram areas for all patient pairs in the evaluation subset. Median histogram intersections were 0.505 (original), 0.739 (AutoRef_F_), 0.738 (AutoRef_FH_), 0.726 (AutoRef_PB_) and 0.724 (Gaussian kernel normalization)

The calculated histogram intersections in Fig. 5 for the 80 cases in the evaluation subset showed that all normalization methods reduced signal intensity variation to a similar extent, with median histogram intersections 0.505 (original), 0.739 (AutoRef_F_), 0.738 (AutoRef_FH_), 0.726 (AutoRef_PB_) and 0.724 (Gaussian kernel normalization). All AutoRef methods had a median histogram intersection slightly higher than the Gaussian kernel normalization. AutoRef_F_ had the best median performance, shortly followed by AutoRef_FH_.

## Discussion

Signal intensity normalization is necessary to reduce heterogeneity in T2WI to enable inter-patient comparison and quantitative analysis of the images, especially between images from different institutions and scanners. This is paramount for the development of computer aided diagnosis of prostate cancer based on mpMRI [11]. AutoRef is a fully automated normalization method recently developed, utilizing fat and muscle as reference tissues (AutoRef_F_) [20]. It is openly accessible^1^ and available to all MRI centres as it requires no additional MRI acquisitions. In the current study, AutoRef was evaluated with three pairs of reference tissues, where AutoRef_PB_ and AutoRef_FH_ were new methods. No significant differences were found between AutoRef_FH_ pseudo-T2s and MESE T2s in all prostate zones for seven volunteers, showing that AutoRef pseudo-T2s can accurately represent measured T2s. Inter-patient variations in prostate intensities in the multicenter dataset decreased with all AutoRef versions, and all four reference tissues under investigation were stably detected in the multicenter cohort. Mann–Whitney *U* test showed that AutoRef reduces significant differences in prostate intensities between scanner pairs.

### Accuracy of reported T2 relaxation times

T2 mapping can be achieved with a range of MR sequences. A set of spin echo (SE) pulse sequences acquired with varied TEs is regarded as the most basic T2 mapping technique [33], but requires long acquisition times in the order of tens of minutes [27]. In addition to affecting patient comfort, this makes the scans prone to motion artifacts [27], diffusion effects [34], chemical exchange [35] and J-coupling [36]. MESE pulse sequences such as the CPMG sequence [37] are typically used to measure T2 for clinical applications with scan time constraints [38]. These sequences sample multiple TE points along the T2 decay for each k-space line during a single repetition time, leading to significantly shorter scan times [27] and a significant reduction of the diffusion effects [27, 34, 38]. They are, however, subject to perturbations from B1 + and B0 inhomogeneities causing strong signal contamination with stimulated and indirect echoes [27, 38], and have shown considerable inaccuracy in the estimation of T2 [39, 40]. The signal contamination yields asymmetry in signal amplitude between odd and even echoes in the echo train, and can to some extent be adjusted for by only including the even echoes in analysis [27, 41, 42]**.**

For the phantom experiments in Online Resource 2, the even-echo analysis of the MESE sequence resulted in measured T2 relaxation times close to the known T2s. However, MESE was observed to overestimate samples with T2 below 148 ms (with root mean square error of 4.1 ms for samples with T2 ∈ [30 ms, 148 ms]), and increasingly underestimate samples with T2 above 148 ms. All tissue T2s measured in this study were below 148 ms, and the phantom results thus indicated that the MESE T2 might be slightly overestimated for these tissues.

The prostate T2s found in this study were similar to what others have reported at 3T [18, 39, 43]. For the reference tissues, Bojorquez et al. [40] found in their literature review large variations in reported tissue T2s at 3T. Fat was reported to be between 41 and 371 ms, bone marrow between 40 and 160 ms and muscle between 27 and 44 ms, suggesting that consensus on reference T2s is not yet established [40]. The reference tissue T2s reported in this work were within the intervals reported in [40]. The low standard deviations observed between subjects in this work indicate that the large variation in T2s observed in the literature is mostly a result of measurement protocol.

Common denominators for the listed studies, including this work, are a limited number of asymptomatic volunteers and a relatively young study population, possibly leading to biased prostate and reference tissue T2s. Younger men have been shown to exhibit lower T2W signal intensity in normal prostatic peripheral zone [44], and the average T2 of the whole prostate is expected to change with age due to extension of the transitional zone due to benign prostatic hyperplasia [2]. The T2 of various muscles has in particular been shown to increase with ageing [46–48], while the literature is scarce on age related changes in T2 for ischioanal fossa, ischial tuberosity and femoral head. Confirmation of the T2s in a multicenter, multivendor clinical cohort would consequently be the next necessary step.

### Comparison of pseudo-T2s to MESE T2

The AutoRef pseudo-T2 of the prostate was evidently dependent on choice of reference tissue. The AutoRef normalization equation (Eq. 2) is expected to overestimate the real T2, as shown in the phantom experiments in Online Resource 2, where overestimation increases with T2 of the high-intensity reference tissue. AutoRef_FH_ is, therefore, in the simplified phantom experiment expected to give the most overestimated pseudo-T2, and AutoRef_PB_ is expected to provide the pseudo-T2s closest to the real T2. However, AutoRef_FH_ pseudo-T2s came closest to measured T2 in volunteers, which substantiates the assumption that the MESE sequence can overestimate T2.

As the pseudo-T2 variation between the three AutoRef methods is somewhat systematic, it could potentially be adjusted for by modelling an expected pseudo-T2 based on reference tissues and scanner parameters such as TE and TR. The pseudo-T2 modelling in the phantom experiments in Online Resource 2 was based on the simple spin echo sequence, and thus only an approximation to the accelerated T2W sequences used to acquire prostate MRI in a clinical setting. In the multicenter evaluation set, an accurate adjustment would require detailed knowledge on how T2WI signal intensities depend on variations in protocols and scanner parameters. These dependencies were not investigated in this work.

### Multicenter evaluation

The reference tissue object detectors succeeded in stably detecting all reference tissues across the multicenter cohort. The pelvic bone detector succeeded in all cases, while the highest proportion of failed cases were seen for the fat and femoral head detectors, with only 1.5% failed cases. AutoRef is, therefore, expected to work for most T2WI. When including cases from the CGMH cohort, however, only cases with an accepted automated prostate segmentation were included. This led to an exclusion of 28% of the available cases, which may have led to a loss of heterogeneity in the multicenter dataset. Including poor segmentations would, on the other hand, give inaccurate prostate intensities and prohibit inter-patient comparisons.

Based on this study, it cannot be concluded which AutoRef version provided prostate pseudo-T2s closest to the ground truth for the multicenter dataset, as the prostate T2s in this cohort were not measured. Based on the results from the volunteers, AutoRef_FH_ is expected to provide pseudo-T2 closest to MESE T2, and thus appeared to be the best choice of reference method with median histogram intersections on par with the best performing AutoRef_F_.

When comparing overall mean multicenter pseudo-T2s to the volunteer pseudo-T2s, AutoRef_F_ and AutoRef_FH_ yielded higher mean pseudo-T2s in the multicenter cohort than for volunteers, even for the Skyra MR system, which was also used for the volunteer study. AutoRef_PB_, however, yielded similar mean pseudo-T2 in both cohorts. The variation in pseudo-T2 could originate from biological variations that should be expected, or be due to the age difference between volunteers and patients, as both the prostate and reference tissues undergo changes with age that can affect MRI signal intensity [2, 45].

### Limitations

There are other potential reference tissues available than the ones chosen for this work. Using the urinary bladder as reference tissue has been shown to improve the performance of T2WI signal intensity for differentiation between prostate cancer and normal tissue [49], but the bladder was not a suitable reference tissue in this study as the T2 relaxation time of urine was too long to measure with our MESE pulse sequence. In addition, the various shapes and sizes of the bladder made it challenging to detect automatically. Other promising reference tissues are the pubic symphysis, gluteus maximus muscle, obturator externus muscle and the body of the pubis. However, these tissues are of similar T2WI intensity as the four reference tissues already under investigation. As AutoRef with these four tissues performed well, it was not deemed necessary to investigate more tissues.

A vast number of parameters could be fine-tuned in AutoRef to enhance performance. In this work, most parameters were the same as, or close to, those reported in [20]. The parameters for training the reference tissue detectors (such as number of training stages) and extraction of ROIs (morphological opening structure, number of evaluated slices) were identical for all reference tissues, apart from focus regions for the reference tissue detectors. It is likely that the various tissues would benefit from varying AutoRef parameter settings, and a systematic optimization of the pre- and post-processing settings on a validation set could be attempted to further enhance performance.

The diagnostic potential of image normalization with AutoRef has not been investigated in this study. The difference in T2 between prostatic carcinoma and healthy tissue has in other studies been reported to be between 30 and 49 ms in the peripheral zone [14, 15, 50] and 9–11 ms in the transitional zone [15]. This indicates that the AutoRef pseudo-T2s might be used for separation of cancer tissue, as the reported T2 variation between healthy and malignant tissue is larger than the mean absolute difference between MESE T2 and pseudo-T2s. In [20], it was in addition shown that applying AutoRef_F_ resulted in a significantly higher area under the receiver operating characteristic curve (AUC) for classification of histologically verified malignant lesions versus healthy prostate tissue, compared to the original un-normalized T2WI. A similar assessment of AutoRef_FH_ and AutoRef_PB_ could be subject for further research.

## Conclusion

In conclusion, reference tissue and prostate T2 relaxation times were measured in asymptomatic volunteers with satisfactory accuracy. All reference tissues under investigation were successfully detected automatically in most cases (96.5%) of the multicenter T2W MRI, and all AutoRef versions succeeded in reducing inter-patient variability. In the volunteer study, only AutoRef_FH_ provided pseudo-T2s showing no significant difference to the MESE T2s. Its ability to standardize multicenter data was comparable to AutoRef_F_, the best performing method in this study. AutoRef_FH_ can, therefore, be considered the best choice for normalization of T2W images of the prostate.

## Supplementary Information

Below is the link to the electronic supplementary material.

Supplementary file1 (PDF 54 KB)

Supplementary file2 (PDF 381 KB)

## Footnotes

1 Publicly available on GitHub: https://github.com/ntnu-mr-cancer/AutoRef.
