# Supplementary material for: Pseudo-T2 mapping for normalization of T2-weighted prostate MRI
Source: MAGMA. 2022 Feb 12;35(4):573–85. doi: 10.1007/s10334-022-01003-9 (PMC9363383; doi:10.1007/s10334-022-01003-9)
Supplement: Supplementary file 1 — Supplementary file1 (PDF 54 KB) [file 10334_2022_1003_MOESM1_ESM.pdf]

Supplementary material 1

Elastix image registration parameters

Registration: Multi-resolution registration
Interpolator: B-Spline
Resample interpolator: Final B-Spline
Fixed image pyramid: Smoothing
Moving image pyramid: Smoothing
Optimizer: Standard gradient descent
Transform: Euler transform
Metric: Advanced mattes mutual information
Automatic scales estimation: Yes
Automatic transform initialization: Yes
How to combine transforms: Compose
Number of histogram bins: 32
Erode fixed mask: No
Erode moving mask: No
Number of resolutions: 4
Fixed kernel B-spline order: 3
Moving kernel B-spline order: 3
Fixed limit range ratio: 0
Moving limit range ratio: 0
Maximum number of iterations: 2048
Number of spatial samples: 2000
Required ratio of valid samples: 1.0000e-03
New samples every iteration: Yes
Image sampler: Random coordinate
Sample region size: [50 50 50]
SP_a: 2000
SP_A: 200
SP_alpha: 0.6000
B-Spline interpolation order: 1
Fixed image B-spline interpolation order: 1
Final B-spline interpolation order: 3
Default pixel value: 0
