# Supplementary material for: Pseudo-T2 mapping for normalization of T2-weighted prostate MRI
Source: MAGMA. 2022 Feb 12;35(4):573–85. doi: 10.1007/s10334-022-01003-9 (PMC9363383; doi:10.1007/s10334-022-01003-9)
Supplement: Supplementary file 2 — Supplementary file2 (PDF 381 KB) [file 10334_2022_1003_MOESM2_ESM.pdf]

Online Resource 2

Phantom study

A phantom study was performed to validate the T2 relaxation times obtained with the multi-echo spin echo (MESE) sequence, and to investigate the relationship between T2 relaxation times and signal intensities in a T2-weighted image (T2WI).

Methods and materials

The phantom was made with ten 50 ml plastic test tubes with different concentrations of manganese(II) chloride tetrahydrate ($MnCl_2 \cdot 4H_2O$), where the sample T1 and T2 relaxation times depend on this concentration [1]. The ten samples had concentrations corresponding to T2s of 283, 217, 169, 148, 129, 109, 89, 69, 50 and 30 ms (named chemical T2) and T1s of 1891, 1685, 1490, 1383, 1280, 1156, 1012, 846, 656 and 428 ms. A 3D printed insert held the ten samples upright within a plastic container filled with NaCl solution to generate a distinct background upon imaging and to inhibit bacterial growth.

MR images were acquired for the phantom on a Magnetom Skyra 3T MRI system (Siemens Healthineers, Erlangen, Germany) at St. Olavs hospital, Trondheim University Hospital, Norway. The phantom was placed upright in a head coil upon scanning, and was supported with foam pads to keep in place. Coronal MESE (TR: 2200 ms, number of slices: 10) and T2W images were acquired with the same sequences as for the asymptomatic volunteers. ROIs were manually drawn within the samples on both the MESE and T2W image using ITK-SNAP [2], as seen in Fig. A1. ROIs were drawn on all image slices, besides the first and last slice as these showed higher relative signal intensity than the other slices. The sample edges were avoided to circumvent Gibbs ringing artifacts. Sample T2 was measured as described in the methods for asymptomatic volunteers, solely using data from the first five even echoes.

The signal intensity in a T2WI can be modelled by the spin-echo solution of the Bloch equations

$$I = k\rho\left(1 - e^{-\frac{TR}{T_1}}\right) \times e^{-\frac{TE}{T_2}}, \qquad \text{(A1)}$$

where k is a proportionality constant dependent on the sensitivity of the signal detection circuitry on the scanner and ρ is the spin (proton) density [3]. As the T2WI in this study were acquired with a turbo spin-echo sequence, and not a simple spin-echo sequence, this can only be regarded as an approximation. By inserting this expression into the AutoRef equation (Eq. 2), an approximation to the expected pseudo-T2 can be achieved. By assuming equal proton densities in the phantom samples (which mainly consist of water), this expression is dependent only on echo time (TE), repetition time (TR) and T1 and T2 relaxation times specific to each sample, in addition to the references used for normalization:

$$\text{pseudo-}T2 = \frac{\left(1-e^{-\frac{TR}{T_1}}\right)e^{-\frac{TE}{T_2}} - \left(1-e^{-\frac{TR}{T_1^{\text{low}}}}\right)e^{-\frac{TE}{T_2^{\text{low}}}}}{\left(1-e^{-\frac{TR}{T_1^{\text{low}}}}\right)e^{-\frac{TE}{T_2^{\text{low}}}} - \left(1-e^{-\frac{TR}{T_1^{\text{high}}}}\right)e^{-\frac{TE}{T_2^{\text{high}}}}} \times \left(T_2^{\text{high}} - T_2^{\text{low}}\right) + T_2^{\text{low}}. \qquad \text{(A2)}$$

Expected pseudo-T2 curves were created for all three AutoRef versions, corresponding to the three reference tissue pairs (AutoRef_F: fat and muscle, AutoRef_{FH}: femoral head and muscle, and AutoRef_{PB}: pelvic bone and muscle), with TE and TR from the T2W pulse sequence used at our institution (TE = 104 ms and TR = 5330 ms).

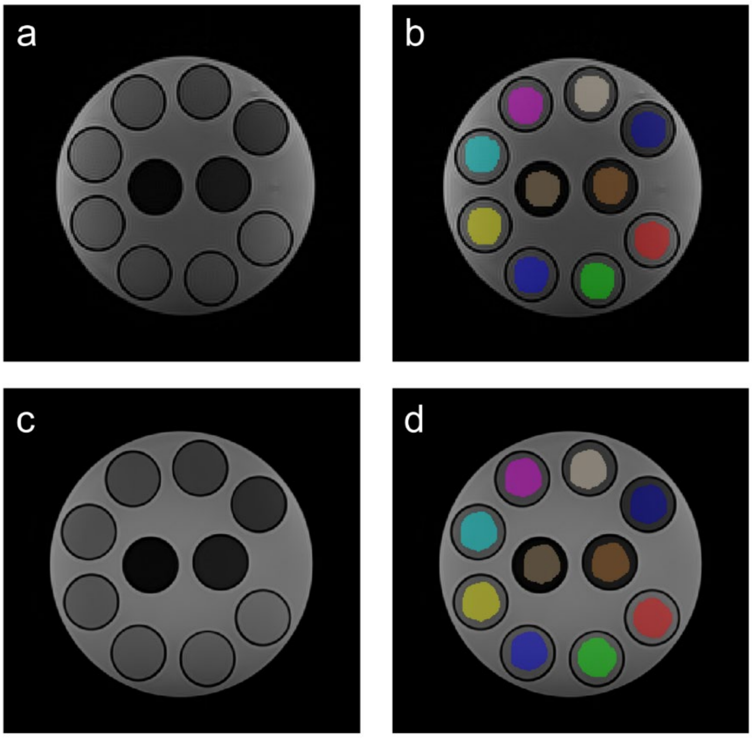

Fig. A1 Coronal multi-echo spin echo (echo time = 106 ms) image of the phantom without (a) and with (b) manual segmentations within the ten samples. c) Coronal T2-weighted image of the phantom. d) Manual segmentations on the phantom T2-weighted image.

A normalization based on AutoRef was applied to the phantom T2WI to investigate the relationship between measured pseudo-T2, expected pseudo-T2 and MESE T2. The modifications made to AutoRef were: The mean ROI intensity was applied instead of the 10th and 90th percentiles, as the delineation of reference ROIs in the phantom was manual. The phantom sample T2s differed from the reference tissue T2s, and therefore the T2WI intensities in the phantom samples were interpolated to match the reference tissues' (fat, muscle, pelvic bone and femoral head) T2s. This interpolation was based on the sample T2WI intensities and chemical T2s, following the equation

$$I^{\text{interpolated}}(T_2) = \frac{I^{\text{low}} - I^{\text{high}}}{T_2^{\text{low}} - T_2^{\text{high}}} \times \left(T_2 - T_2^{\text{high}}\right) + I^{\text{high}}, \qquad \text{(A3)}$$

where high and low refers to the two samples closest to the desired T2 value, and *I* is the mean intensity in a sample. The 3D T2WI was then linearly scaled, as in AutoRef, with the interpolated reference intensities and corresponding T2s.

Results

The MESE T2s (Fig. A2) were within relative error 13% to the phantom chemical T2s, with root-mean-square error (RMSE) of 10.4 ms. For the samples below 148 ms the MESE appeared to give a slight overestimation, while for the samples above 148 ms the MESE T2 was underestimated with

more prominent underestimation with higher chemical T2s. By excluding the three samples with highest T2 the RMSE decreases to 4.1 ms. The tissue T2s measured in this work were all below 148 ms, which in the following is referred to as the relevant region.

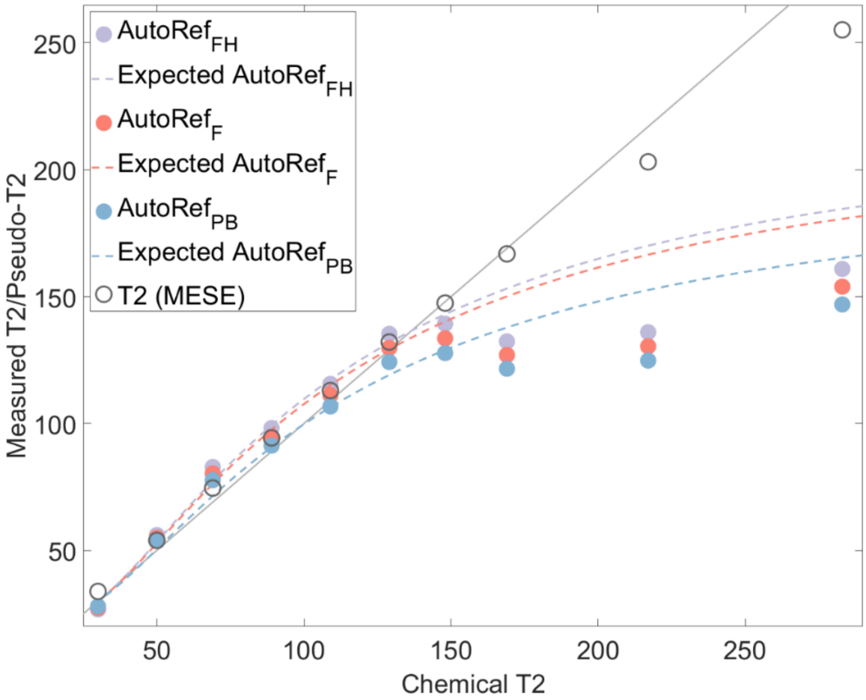

Fig. A2 Normalized T2-weighted signal intensity (measured pseudo-T2) with corresponding expected pseudo-T2, versus chemical T2 in each sample in the phantom. The normalization was executed with the modified version of AutoRef, corresponding to the three AutoRef versions used *in vivo*. T2 relaxation times obtained with MESE for each sample are also included in the figure.

The expected pseudo-T2 curves in Fig. A2 show that all AutoRef versions should give somewhat overestimated pseudo-T2s in the interval between the T2s of the respective reference tissues. Passing the high intensity reference T2, the pseudo-T2 is expected to be underestimated. The measured pseudo-T2s appear to follow this pattern up until 148 ms, after which it becomes considerably lower than the expected pseudo-T2. This deviation could be because the spin echo signal equation is only an approximation to the turbo spin echo sequence actually used, or originate in artifacts induced during acquisition. In the relevant region, AutoRef$_{PB}$ is expected to come closest to the chemical T2, as this version gives the least overestimated pseudo-T2 due to the low T2 of the high intensity reference tissue. As the MESE T2 also appears to be overestimated, AutoRef$_{F}$ and AutoRef$_{FH}$ give expected and measured pseudo-T2s close to the MESE T2. The RMSE between AutoRef versions and MESE in the relevant region were 5.6 ms (AutoRef$_{FH}$), 6.3 ms (AutoRef$_{F}$) and 8.8 ms (AutoRef$_{PB}$).

Discussion

The MESE sequence is designed to circumvent diffusion effects, but the effect cannot be completely diminished. Inhomogeneities in the main static magnetic field or eddy current transients from the gradient coils will induce a diffusion dependency on the MESE measured T2 [4]. This effect will lead to an underestimation of T2 that becomes more prominent in samples and tissues with long T2s and samples with a high degree of diffusion [5]. The relatively large bulk volumes in the phantom samples would likely contain water circulation, e. g. because of mechanical vibrations induced by the gradient pulses or temperature variations in the samples. This can explain the deviation from chemical T2 for

samples with high T2s. The rate of diffusion in tissue in vivo is expected to be lower, and hence the underestimation of T2 is expected to be less prominent in vivo. This is to some extent hindering a direct comparison between the MESE results in phantoms and asymptomatic volunteers, although the phantom results can be used to make assumptions about the volunteer results.

Conclusion

The AutoRef pseudo-T2 is expected to be dependent on choice of reference tissue. Regarding the region of prostate T2, $AutoRef_{PB}$ is expected to give the pseudo-T2 most accurate to real T2, while $AutoRef_{F}$ and $AutoRef_{FH}$ are expected to give a more overestimated pseudo-T2. The multi-echo spin echo measured T2 is expected to be quite accurate in this region, although slightly overestimated.

References

1. Nofiele JT, Cheng H-LM (2013) Ultrashort Echo Time for Improved Positive-Contrast Manganese-Enhanced MRI of Cancer. PLOS ONE 8:e58617.
2. Yushkevich PA, Piven J, Hazlett HC, Smith RG, Ho S, Gee JC, Gerig G (2006) User-guided 3D active contour segmentation of anatomical structures: significantly improved efficiency and reliability. NeuroImage 31:1116–1128.
3. (2014) Spin Density, T1, and T2 Quantification Methods in MR Imaging. Magn. Reson. Imaging. John Wiley & Sons, Ltd, pp 637–667
4. Torrey HC (1956) Bloch Equations with Diffusion Terms. Phys Rev 104:563–565.
5. Oakden W, Stanisz GJ (2014) Effects of diffusion on high-resolution quantitative T2 MRI. NMR Biomed 27:672–680.
